# Coupled membranes: a mechanism of frequency filtering and transmission in the field cricket ear evidenced by micro-computed tomography, laser Doppler vibrometry and finite element analysis

**DOI:** 10.1098/rsif.2023.0779

**Published:** 2024-06-21

**Authors:** Brendan Latham, Andrew Reid, Joseph C. Jackson-Camargo, Jonathan A. Williams, James F. C. Windmill

**Affiliations:** ^1^ Bioacoustics Group, Centre for Ultrasonic Engineering, Department of Electronic and Electrical Engineering, University of Strathclyde, Glasgow, UK; ^2^ Department of Biomedical Engineering, University of Strathclyde, Glasgow, UK

**Keywords:** insect ear, micro-CT, LDV, finite element analysis, auditory system, biomimetic

## Abstract

Many animals employ a second frequency filter beyond the initial filtering of the eardrum (or tympanal membrane). In the field cricket ear, both the filtering mechanism and the transmission path from the posterior tympanal membrane (PTM) have remained unclear. A mismatch between PTM vibrations and sensilla tuning has prompted speculations of a second filter. PTM coupling to the tracheal branches is suggested to support a transmission pathway. Here, we present three independent lines of evidence converging on the same conclusion: the existence of a series of linked membranes with distinct resonant frequencies serving both filtering and transmission functions. Micro-computed tomography (µ-CT) highlighted the ‘dividing membrane (DivM)’, separating the tracheal branches and connected to the PTM via the dorsal membrane of the posterior tracheal branch (DM-PTB). Thickness analysis showed the DivM to share significant thinness similarity with the PTM. Laser Doppler vibrometry indicated the first of two PTM vibrational peaks, at 6 and 14 kHz, originates not from the PTM but from the coupled DM-PTB. This result was corroborated by µ-CT-based finite element analysis. These findings clarify further the biophysical source of neuroethological pathways in what is an important model of behavioural neuroscience. Tuned microscale coupled membranes may also hold biomimetic relevance.

## 1. Introduction

The ear of the field cricket (family Gryllidae, subfamily Gryllinae) is notable for its remarkable sound-source localization accuracy level to that of humans [1]. It exhibits a rare instance among insects of tonotopy, with auditory neurons arranged along a low- to high-frequency gradient [2]. Approximately 70 sensory neurons [3] are individually tuned within frequency clusters, including those matching the carrier frequencies (CFs) of the calling and courtship songs [4], produced by the male’s forewings [5]. Some neurons are tuned to the courtship song CF [4], between 11 and 16 kHz in *Gryllus bimaculatus* [6], while most are sharply tuned to the 4–5 kHz dominant frequency of the calling song [4,7].

The distinctive [8,9] pure-toned calling song is essential for facilitating reproduction in field crickets. Before mating, females must locate the male’s position from a distance, and studies have shown this localization behaviour is highly dependent on the CF of the conspecific calling song [1,10]. Electrophysiological recordings of individual receptors [2], the whole tympanal nerve [11] and the AN1 interneuron [12] all demonstrate acute selectivity to this frequency. This predictability in phonotactic behaviour, achieved via identifiable neural networking, has made the field cricket a valuable neuroethological model [9]. Thus, behaviour and neurophysiology are established domains in the 4–5 kHz sharp tuning of the field cricket ear. However, how the peripheral auditory anatomy causes this frequency filtering has long been acknowledged as unresolved [13–20].

**Figure 1.**
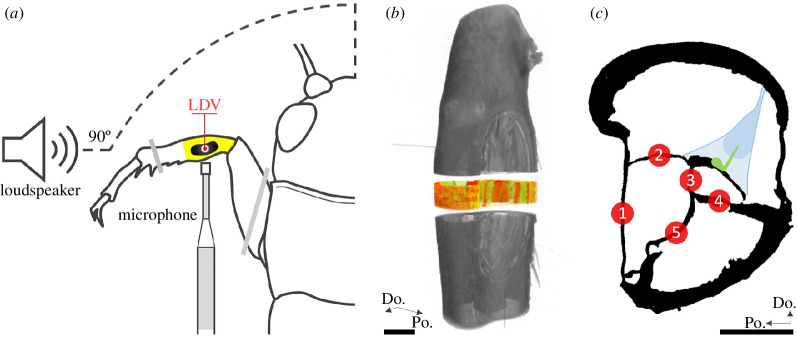
LDV set-up and µ-CT thickness analysis. (*a*) Schematic of LDV experimental set-up (see also electronic supplementary material, figure S2). The loudspeaker was level with the cricket 22 cm from its midline and 90° ipsilateral. The reference microphone was perpendicular to the loudspeaker within 1 cm of the ipsilateral tympanum. (*b*,*c*) Methodology employed in µ-CT 3D thickness analysis. (*b*) Volumetric visualization illustrating the approximate position of the image stack at the midpoint of the PTM from which the five cylindrical samples of diameter 20 × 55 µm were segmented. (*c*) The five structures (1–5) that were sampled and the positions in the middle of each structure from which the cylindrical segmentation was taken: 1. PTM; 2. DM-PTB; 3. DivM; 4. VW-ATB; 5. VW-PTB. The illustration in blue represents the tympanal organ and its arrangement based on the literature, e.g. [21]; green = sensory neurons. PTM, posterior tympanal membrane; DM-PTB, dorsal membrane of the posterior tracheal branch; DivM, dividing membrane; VW-ATB, ventral wall of the ATB; LDV, laser Doppler vibrometry. Scale bars*:* 200 µm.

The field cricket possesses two ears, one in each of the two front legs, at the proximal end of the tibia leg segment (see figure 1*a*, yellow highlight). Each ear has two tympana. The smaller and thicker anterior tympanal membrane (ATM) has been considered negligible for both reception and filtering [10,17,19], while the posterior tympanal membrane (PTM) is regarded as the primary sound receiver [10,16,19,20,22].

Unlike the ears of insects such as moths [23], the field cricket’s sensory neurons are not directly attached to the tympanum. Instead, the PTM and the sensors are separated by two air-filled cavities, the tracheal branches, each with a membranous dorsal wall [18]. Auditory neurons are arranged tonotopically along the dorsal membrane of the anterior tracheal branch (DM-ATB) [2,18], where they are immersed in a lipidic fluid within a complex of structures known as the tympanal organ [18].

**Figure 2. F2:**
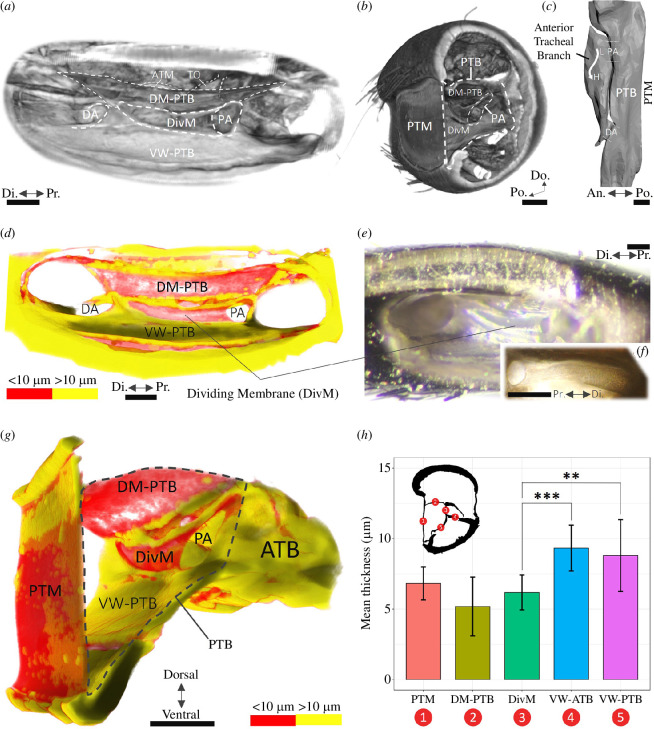
µ-CT visualization and 3D thickness analysis of the field cricket tracheal branches and PTM. (*a*,*b*) µ-CT volumetric visualization. (*c*) Segmentation of the air column. (*d*,*g*) Three-dimensional thickness colour maps (see also electronic supplementary material, animation S1). Cuticle below 10 µm in thickness is coloured red. (*e*,*f*) Photographs of the DivM taken under light microscopy from a left (*e*) and a right (*f*) ear. (*h*) Results of the thickness analysis of the five sampled structures (*n *= 12, s.d. error bars, one-way ANOVA with Tukey’s HSD). Results indicate the DivM is significantly thinner than two of the structures: the VW-ATB (***p<0.001) and the VW-PTB (**p<0.01). (Only significant differences relating to the DivM are shown.) ATM, anterior tympanal membrane; TO, tympanal organ; DivM, dividing membrane; PA, proximal aperture; DA, distal aperture; DM-PTB, dorsal membrane of the posterior tracheal branch; VW-PTB, ventral wall of the PTB. Scale bars: 100 µm.

The field cricket anterior tracheal branch (ATB) is distinct compared to other more arboreal ensiferans: Eneopeterinae [21], Oecanthinae [24], Tettigoniidae [25] and Hagloidea [26]. Unlike these taxa, the Gryllinae ATB is not in direct contact with the ATM and is considerably smaller than the PTB (see figure 2*c*). Two small holes or 'apertures' connect the branches, the proximal aperture (PA) and the distal aperture (DA) (see figure 2 and electronic supplementary material, animation S1).

The larger PTB is more of a continuation of the leg trachea [18], part of the wider acoustic trachea connecting the two ears and two acoustic spiracles [17,27]. The spiracles are openings on either side of the thorax that provide two major sound inputs through the acoustic trachea to each ear [27]. The signal from the contralateral spiracle passes through the ‘medial septum’ in the middle of the animal which subjects the sound wave to an additional phase shift [28,29]. The contralateral and ipsilateral sound waves then superimpose before reaching the PTM from inside the posterior branch [14]. As such, the large tympanum is stimulated not only externally but also from a phase-shifted signal acting on its internal surface [14,30]. However, reports disagree on how these tympanal vibrations relate to the tuning of the ear to the calling song CF [14,19,21,30–32].

Several studies have reported a sharp vibrational response of the posterior tympanum close to the CF of the calling song [15,31,32]. For instance, in 1981, a distinct PTM velocity peak at 5.5 kHz was recorded [15]. A review in 2016 noted a close correspondence of this finding with the tuning curve of the tympanal nerve, and the PTM was therefore implicated as a sufficient biophysical filter determining the CF-tuning of the auditory pathway at the neural and behavioural levels [33]. Similarly, Johnstone *et al*. [31] observed a single displacement optimum at 4.0 kHz that indicated the PTM *'is mechanically tuned to a relatively narrow spectrum of frequencies*'.

A strong *direction-dependent* tuning of the PTM to the calling song CF has also been evidenced. By isolating each of the sound inputs, Michelsen *et al*. [27] calculated a significant phase shift when the calling song frequency was approached. This supported an earlier phase-shift hypothesis [13,29], whereby the internal–external phase difference exaggerates motion of the ipsilateral PTM, thus enhancing directional cues at that frequency. Experimental evidence was provided by Michelsen & Löhe [32], of a pronounced PTM peak close to the calling song CF when the sound source was presented ipsilaterally. Together, these findings suggest the PTM is indeed sharply tuned to the CF of the calling song, either from mechanical resonance and/or directional tuning.

However, other early measurements of the PTM show a CF optimum which is nonetheless too broad to match the tight tuning curves of the mechanosensory neurons [17,19]. A second frequency filter has, therefore, long been conjectured [16,17,20], and the speculated filter has even been compared to that of the mammalian cochlea [17].

More recent recordings suggest a PTM optimum as high as 11–17 kHz with broad filtering [21]. Further testing of the phase-shift hypothesis does support directional tuning [14,30] but reveals a broader response than observed by Michelsen & Löhe [32] and even at optima higher than the calling song CF [14]. The proposed second filter, situated between the posterior tympanum and the sensory neurons, has therefore received renewed support [14]. Yet the anatomy of this region is complex [3,18] and how PTM vibrations are communicated to the sensors has remained *'enigmatic*' [18].

Nevertheless, the well-known apposition of the posterior PTB wall against the large tympanum is thought to be a likely means of mechanically coupling tympanal vibrations to the tracheal branches [16,18,21,22]. The arrangement of the branches beside each other probably provides a connection from the PTM to the anterior branch supporting the neurons [21]. Moreover, the dorsal membrane of the larger branch is known to connect directly to the PTM [18]. Given this morphology, Nishino *et al*. [18] in their 2019 study propose two possible transmission routes. One involves PTM coupling to the DM-PTB affecting the fluid surrounding the sensilla, while the other involves air movement through the connecting apertures causing inflation of the ATB. It is not known whether either of these pathways is in effect [18].

As such, (i) not only is the existence of secondary filtering hypothesized, but (ii) the path of transmission also remains to be identified. Accordingly, we aimed to address both research gaps using three separate methods: micro-computed tomography (µ-CT), laser Doppler vibrometry (LDV) and finite element analysis (FEA).

Our µ-CT results revealed the presence of the ‘dividing membrane’ (DivM) separating the branches and linking to the PTM via the DM-PTB. Three-dimensional (3D) analysis indicated that the DivM shares significant thinness similarity with these two membranes, further suggesting its auditory functionality. LDV recordings confirmed the existence of at least two distinct PTM vibrational peaks: a natural resonance around 14 kHz and a 6 kHz peak derived externally, probably from DM-PTB tuning. The µ-CT data served as the basis for an FEA model, which supported the LDV findings and indicated a series of tuned membranes including the DivM and the DM-ATB beneath the sensors. Numerical modelling also suggested the possibility of a membrane-mediated volume change of the ATB. We discuss how these findings contribute to understanding filtering and transmission in this remarkable auditory system.

## 2. Methods

### 2.1. Animals

Mediterranean field crickets (*Gryllus bimaculatus*, De Geer) were commercially sourced and used for µ-CT and FEA analyses (supplier: Blades Biological Ltd, UK), and as the primary subject of LDV experiments (local supplier: Pets At Home, Glasgow). In addition, the Australian species *Teleogryllus commodus* were used for LDV tests and were provided from a lab colony (courtesy of Nathan Bailey, University of St Andrews, UK) originating from wild caught females (near Moss Vale, New South Wales).

Crickets were kept in a 12 : 12 h light : dark cycle, fed ad libitum, and given egg-carton sheltering. *G. bimaculatus* were placed as adults in an incubator (OVA-Easy 190 Advance, Brinsea) at 26°C within 15 l plastic containers and fed fish-food flakes, organic wheatgerm and gel-water. *T. commodus* were bred in a temperature-controlled room at 25°C within 16 l plastic boxes, with rabbit food and water-soaked cotton pads.

**Figure 3. F3:**
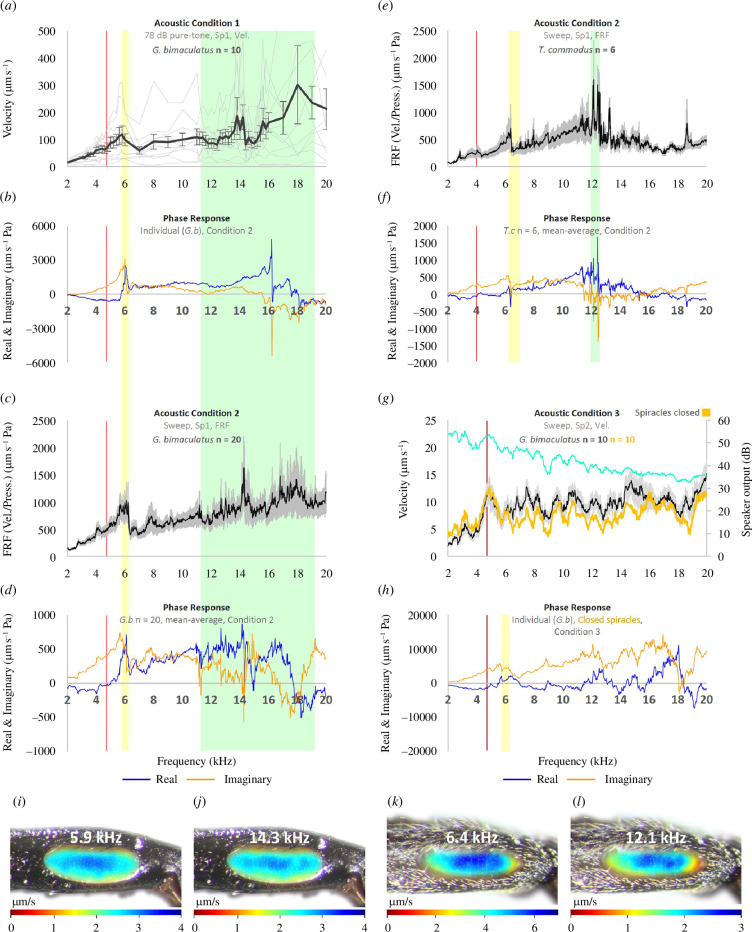
Responses of the field cricket PTM from LDV experiments. (*a*) Pure tone velocity recordings taken with a constant sound pressure level of 78 dB and indicating two vibrational peaks, one at 5.8 kHz and a second at 13.8 kHz (*n *= 10, mean ± s.d.). (*b–f*) Recordings from frequency sweeps (see electronic supplementary material, figure S3 for loudspeaker outputs). (*b*) The phase response of a representative tympanum showing the PTM vibrated with a driving-force at 6.0 kHz (yellow highlight) and a natural resonance at 16.2 kHz (green highlight). (*c*) Mean-average (*n *= 20, ± s.e.m.) FRF of PTM velocities in response to sound pressure, showing a vibrational optimum at 6.0 and 14.3 kHz. (*d*) Mean phase (*n *= 20, ± s.e.m.) revealing the 6.0 kHz peak was a driving-force and the 14.3 kHz peak the natural resonance of the tympanum. (*e,f*) PTM response from *T. commodus* (*n *= 6, mean ± s.e.m.) showing a driving-force peak at 6.4 kHz and the natural resonance at 12.2 kHz. (*g,h*) PTM velocities with the spiracles *open* (black, *n *= 10, mean ± s.e.m.) and *closed* (orange, *n *= 10, mean) using a ribbon-diaphragm AMT loudspeaker (loudspeaker output shown). The data were noisy, but a corresponding phase response (*h*) suggested a maintained driving-force near 6.0 kHz. (*a–h*) The widths of the yellow and green highlights correspond to the ranges in frequency positions across individuals (*G. bimaculatus*, *n *= 20; *T. commodus*, *n *= 6). (*i–l*) Maximal velocity per LDV scan point at peak 1 and 2 in *G. bimaculatus* (*i,j*) and *T. commodus* (*k,l*) showing the PTM oscillated with a drum mode at both peaks (see also electronic supplementary material, animation S5). *G*.*b, G. bimaculatus; T.c*, *T. commodus*; FRF, frequency response function; AMT, air motion transformer.

The CF of the *G. bimaculatus* calling song is 4.7 kHz and that of *T. commodus* is 4.0 kHz [7]. In figures 3, 5–7, the calling song CF is represented by a thin vertical red line.

### 2.2. µ-CT

In identifying anatomical structures of potential auditory functionality, a *thin* cuticle over an air-filled cavity would be of especial relevance [34]. This is because thin structures have less mass and lower stiffness, giving them greater vibrational displacement and therefore greater sensitivity to acoustic waves [35].

#### 2.2.1. Insect preparation

Insects were killed by ethyl acetate fumigation [36]. The portion of the tibia containing the ear was excised with microscissors and submerged in 1:1 alcoholic Bouin’s solution [37] in an Eppendorf tube and fixed overnight at 23°C. Fixative was extracted and the specimen was washed three times in 70% ethanol for 10 min each time, before passing through an ethanol dehydration series (10 min in solution, 10 min air-drying at room temperature: 50%, 70%, 80%, 90%, 95%, and three cycles of 100%). Tibial sections were submerged in 0.3% phosphotungstic acid (PTA) stain for optimal contrast [37] and kept at 23°C for three nights. The PTA solution was removed, and the specimen was rinsed in 100% ethanol (10 min, three cycles) and then finally chemically dried by immersion in 100% hexamethyldisilazane for 2 h before air-drying at 35°C overnight [38]. Specimens were stored in 70% ethanol prior to µ-CT scanning.

#### 2.2.2. Imaging, segmentation and thickness analysis

Tibial sections were mounted vertically on a brass sample rod and imaged in air using a Bruker SkyScan 1172 µ-CT scanner. All scans were performed with an exposure time of 1325 ms, 0.2° rotation steps for 180°, a frame averaging of 2, a current of 100 µA and without a metal filter.

Thickness measurements (figures 1*b,c* and [Fig F2]2[Fig F2] *h*) were obtained from 12 individuals (six female, six male) scanned with an X-ray tube voltage of 48 kV and an isotropic voxel size of 0.55 µm. Membrane and air column FEA models were derived from a single representative specimen (female) scanned at 30 kV and 0.88 µm voxels (figures 5–7 and electronic supplementary material, animation S1). Projection images were reconstructed using Bruker NRecon software (v. 1.6.9.18).

The thickness relationships of the DivM with surrounding structures were quantified (figur[Fig F2]e 2*h*) within cylindrical volumes of interest (diameter 20 µm, height 55 µm) located at the midpoint of the PTM (figure 1*b*). Five structures were analysed (see figure 1*c*): the PTM, the DM-PTB, the DivM, the ventral wall of the ATB (VW-ATB) and the VW-PTB. The DM-ATB was not segmented as the membrane could not be differentiated from adhered non-DM-ATB material. The five samples were binarized by global threshold (21–255) and denoised (removal of all except largest object, in 3D). Thickness was then determined in CT Analyser (v. 1.14.10.0) using the maximal diameter sphere-fitting technique [39].

Transmission [18,29] and filtering [19] have been hypothesized to involve the air column inside the tracheal branches, rather than solid–solid tracheal coupling. As such, the air column too was segmented (figures 2*c* and 7*c*). Segmentation was performed in 3D Slicer (v. 4.11.20210226) with the threshold paint tool. The mesh was cleaned in MeshLab (v. 2021.07) before exporting as an STL file for subsequent FEA simulation.

### 2.3. Laser Doppler vibrometry

#### 2.3.1. Set-up

The vibrational response of the posterior tympanum was recorded from a total of 41 *G. bimaculatus* and six *T. commodus* using a 3D LDV (MSA-100-3D, Polytec) (figure 3). To anaesthetize, animals were chilled in a freezer for 5 min at −18°C. Spiracle flaps were then removed with microscissors or blocked with petroleum jelly (Vaseline^®^). The cricket was pinned to a wax bed upon a goniometer (GN1/M, Thorlabs) attached to an aluminium platform custom-built to screw into the LDV X-Y position stage. Posterior tympana were secured facing upwards and the anterior tympana were kept free from underlying wax (see figure 1*a* and electronic supplementary material, figure S2).

The LDV sat upon a vibration isolation air table inside a semi-anechoic double-walled audiometric room (IAC Acoustics). The loudspeaker was situated level to the animal, 22 cm from the cricket’s midline, at 90° azimuth, ipsilateral to the target PTM. To provide a reference signal, a 1/8″ precision pressure microphone (Type 4138 A-015, Brüel & Kjær), connected to a conditioning amplifier (Type 2690 A-0F2 NEXUS, Brüel & Kjær), was positioned perpendicular to the loudspeaker within 1 cm of the ipsilateral tympanum (figure 1a and figure S2).

#### 2.3.2. Stimuli

LDV experiments were conducted under three acoustic conditions. All stimuli were presented at 2–20 kHz with 1600 fast Fourier transform lines and averaged over five cycles of complex averaging.

##### 2.3.2.1. Acoustic condition 1

Pure tones (sine waves) were presented through a 5.8 cm diameter cone-diaphragm loudspeaker (VISATON FR58, 8 Ω, 120–20,000 Hz) at a linear sound pressure output of 78 dB [14] to crickets with open spiracles (flaps cut) (figure 3*a*). Single point measurements were obtained from the middle of the tympanum in 10 *G. bimaculatus* (five male, five female). Experimental duration was optimized: field crickets communicate using three songs [11], each of which has a dominant frequency between either 3–6 kHz [8] or 11–16 kHz [6]. As such, these frequency ranges were measured in 200 Hz bins; all other frequencies were binned every 1000 Hz. Results are presented as velocities (µm s^−1^).

##### 2.3.2.2. Acoustic condition 2

Sweeps (periodic chirps) applied through the same loudspeaker provided multipoint measurements of the whole tympanum at finer 15.625 Hz frequency bins (figure 3*b–f*). Recordings were taken from 20 *G. bimaculatus* (10 female, 10 male) and six *T. commodus* (one female, five male), with spiracles open, using a manually drawn outline of the PTM. Within the outline, a mesh of scan points was autogenerated with distances between points of app. 72 µm. To control for any nonlinearity in the loudspeaker outputs (see electronic supplementary material, figure S3), results are presented as the frequency response function (FRF) of velocity in response to sound pressure (µm s^−1^ Pa).

##### 2.3.2.3. Acoustic condition 3

Sweep recordings were also taken with a second loudspeaker (figure 3*g,h*) that uses a ribbon-diaphragm technology, known as a Heil air motion transformer (ESS, 800-20,000 Hz). PTM responses were recorded from 10 *G. bimaculatus* (six female, four male) with closed spiracles and from a further 10 (six female, four male) with spiracles open. Results are presented as velocity (µm s^−1^) alongside the mean output of the loudspeaker (dB).

### 2.4. Finite element analysis

#### 2.4.1. Demonstrating coupled resonators

**Figure 4.**
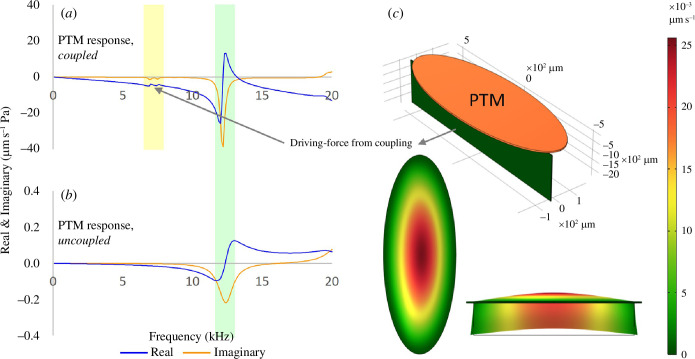
FEA demonstration of a coupled resonator driving the first PTM peak. (*a,b*) The driving-force (yellow highlight) was only evident when the PTM was coupled. (Green highlight = natural resonance of the membrane.) (*c*) FEA model in coupled configuration (external resonator in dark green).

LDV revealed the 6 kHz peak to be a driving-force rather than the natural resonance of the tympanum, which was identified at around 14 kHz (see figure 3). To demonstrate that this experimental finding can be reproduced by the action of a mechanically coupled resonator, the PTM was simulated in COMSOL Multiphysics^®^ (v. 6.1) *with and without* its DM-PTB coupling (see figure 4).

The PTM was represented by a 1083 × 397 µm two-dimensional (2D) ellipsoid primitive with a thickness of 8 µm. When coupled, a 240 × 1100 µm shell element was attached as the DM-PTB, 50 µm above the tympanum’s major axis, and assigned a thickness of 4 µm. Coupled–uncoupled simulations were swept over the frequency domain from 1 to 20 kHz in 100 Hz steps. Instantaneous velocity was calculated from the tympanum in isolation and when coupled before conversion into real and imaginary values (µm s^−1^ Pa) for standard modal analysis.

#### 2.4.2. Simulating the tracheal branches

**Figure 5. F5:**
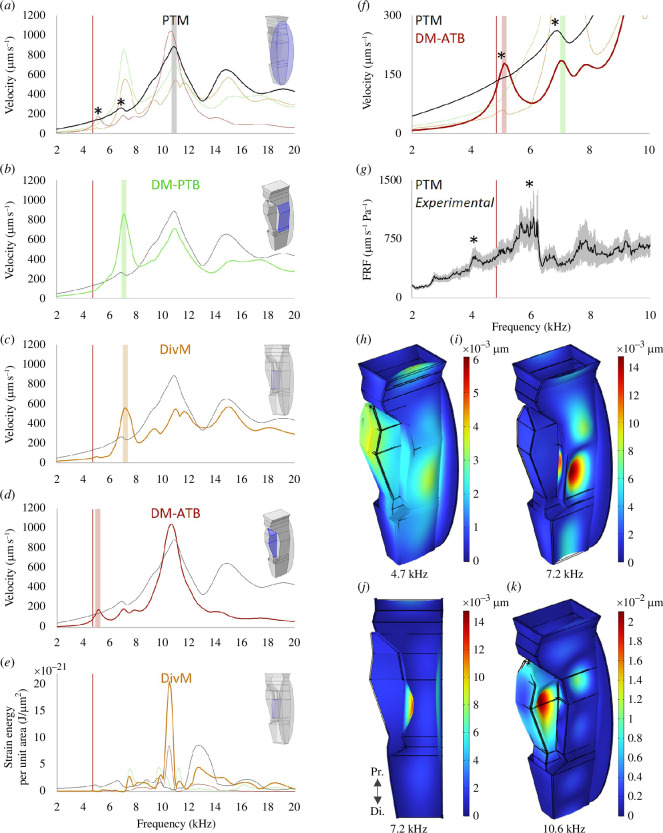
Vibrations of the internal membranes and PTM according to FEA. (*a*) The velocity profile of the PTM exhibited a robust match with experimental results (see figure 3) in showing a main resonance at 10.7 kHz with a lower-frequency peak at 7.2 kHz that (*b*) exactly matched the dominant resonance of the internally connected DM-PTB. (*c*) The DivM resonated with a peak velocity of 7.0 kHz. (*d*) The dorsal membrane of the ATB, which supports the sensory neurons, responded with a distinct resonance closer to the calling song CF. (*e*) The DivM effectively amplified the vibrational energy transmitted from the PTM, at the tympanum’s resonant frequency. (*f*) A very slight but nonetheless evident disturbance of the PTM was directly correlated with the resonant frequency of the DM-ATB (left-hand star), (*g*) which conceivably corresponds with a possible subpeak in the LDV-recorded response. (*h–k*) The µ-CT-based FEA model used, showing boundary displacements at relevant frequencies.

Data from µ-CT and LDV supported a resonance function of the DivM and DM-PTB, respectively. These membranes were numerically modelled in COMSOL within a finite-element model of the intact system, constructed from µ-CT data (see figure 5*h-k*).

##### 2.4.2.1. Dimensions from µ-CT

Model dimensions were taken from the 0.88 µm dataset (described in §2.2.2.). Cross-sectional µ-CT slices were segmented by binary-threshold (an example slice can be seen in figure 1*c*). The binarized images were used to outline the branches by placing vertices at the points where the tracheal walls intersect. Given the inherent irregularity of anatomical positions, traces were made on arbitrary slices by visual inspection of significant anatomical features. Outlines were made on 10 irregularly spaced images, covering the full extent of the branches. A total of 63 vertices were then connected by linear interpolation to form a wireframe mesh.

The maximal length and width of the PTM were measured from the same µ-CT dataset using a segmentation of the tympanum in 3D Slicer. The measured dimensions were then assigned as the major and minor axes of a 1083 × 397 µm ellipsoid, positioned coincident with the posterior side of the hexahedral mesh (figure 5*h–k*).

##### 2.4.2.2. Thicknesses from µ-CT

The wireframe was converted to a shell physics model and the thicknesses from µ-CT measurements (see figure 2*h*) were assigned to corresponding elements of the model: PTM, 6.8 µm; DM-PTB, 5.2 µm; DivM, 6.2 µm; DM-ATB, 5.2 µm; non-membrane elements, 9.1 µm. The DM-ATB was given the same thickness value as its corresponding dorsal membrane of the PTB. The thicknesses of all non-membrane boundaries were assigned as the mean average of the two ventral wall thickness values (figure 2*h*).

##### 2.4.2.3. Material properties from the literature

**Figure 6. F6:**
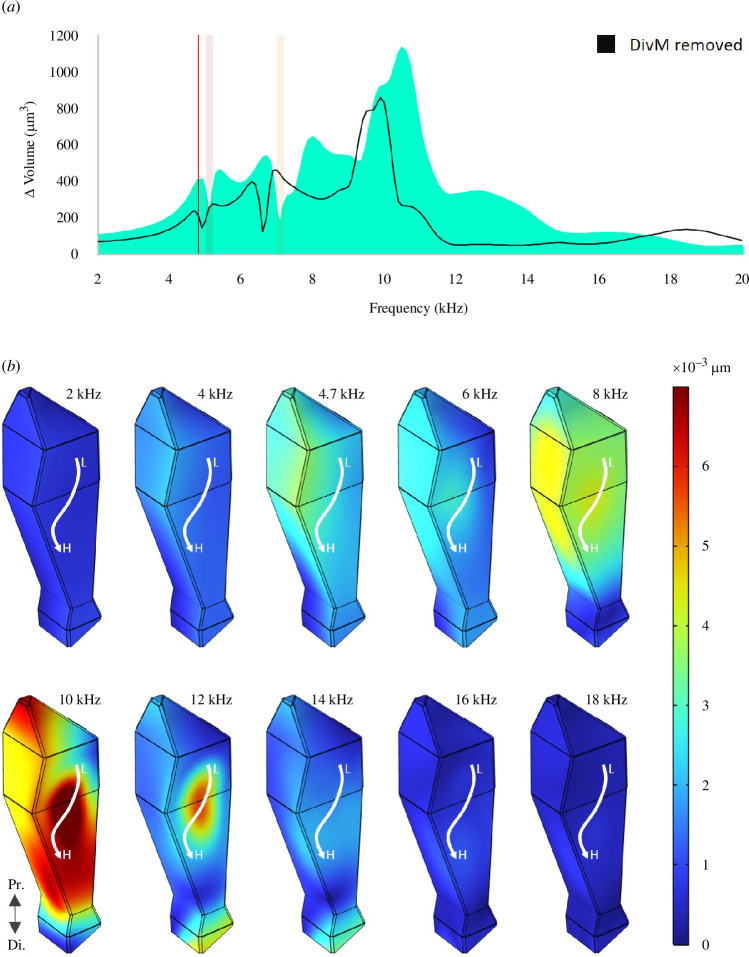
Membrane-mediated volume change of the ATB according to FEA. (*a*) The anterior branch inflated and deflated within a biologically relevant frequency range, between 4.8 and 10.4 kHz. The intermediate nulls were correlated with the membrane resonances (see figure 5). Removing the DivM from the system (black) reduced ATB volume by 24.5% at maximal peak and lowered all spectra by around 600 Hz. (*b*) Mosaic showing ATB boundary displacements at different frequencies. (The rest of the system, including the PTM, is not shown; the DivM was in place.) The ATB exhibited relative deflation at 2 kHz with increasing volume towards 4.7 kHz, then a slight decrease, before expanding to maximal volume around 10 kHz and then deflating towards 18 kHz. The white arrow represents the approximate positions of the low- (L) to high-frequency (H) tuned mechanoreceptors as based on Nishino *et al*. [18].

The material properties of the gryllid PTM and tracheal branches are unknown, and as such were here based on previous FEA modelling of the locust tympanum by Malkin *et al*. [40]. The model was made an isotropic linear-elastic solid and assigned a Poisson’s ratio of 0.3 and a density of 1300 kg m^–3^. The PTM was given a fixed boundary condition and assigned a Young’s modulus of 20 MPa. All non-PTM elements were given a lower value of 2 MPa, in line with the cuticle of the cricket leg trachea known to contain the rubber-like protein resilin [41]. The model was swept over the frequency domain in steps of 100 Hz from 1 to 20 kHz. Maximum velocity of the element (µm s^−1^) and strain energy density at the element’s midpoint (J µm^–2^) were calculated (figure 5). Volume change of the anterior branch has previously been proposed, albeit from aperture airflow [18]. We calculated *membrane-mediated* volumetric increase as the integral of the displacements of all ATB boundaries (µm^3^) (figure 6).

### 2.4.3. The air column

**Figure 7.**
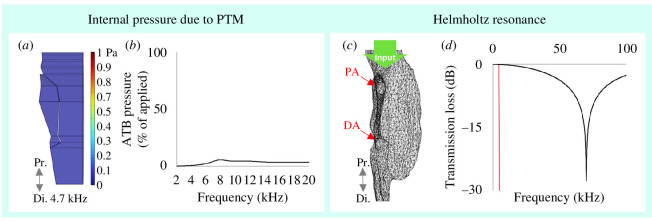
Tympanal driven pressure change and cavity resonance inside the tracheal branches according to FEA. (*a,b*) Air column pressure from stimulating the posterior tympanum with 1 Pa pressure, within (*a*) the FEA shell model. Figure shows tracheal pressure at 4.7 kHz. (*b*) Pressure inside the ATB was less than 10% of that applied from the PTM, indicating the PTM is a negligible influence on internal air pressure. (*c*) µ-CT segmentation of the air column. A sharp loss in pressure below the proximal and distal apertures (PA and DA) relative to the input pressure above (green arrow) was considered indicative of a Helmholtz resonance. (PTM was ignored.) (*d*) The air column of the tracheal branches did not resonate until 70.3 kHz, considerably above the relevant range of communication signals [6,7,11].

The role of the PTM in effecting *pressure change* [18,29] inside the tracheal branches was investigated using the shell physics model (figure 7*a*) and applying 1 Pa pressure to the tympanum. Doing so had little effect on ATB air pressure (figure 7*b*) which indicated movement of the PTM exerts a negligible influence on internal air pressure.

A *cavity resonance* [19] was investigated using the µ-CT segmentation of the air column (figure 7*c*), which was treated as a thermoviscous pressure acoustics model. The proximal opening that leads into the PTB was the sole pressure input (given the PTM was negligible). The morphology of the tracheal branches and apertures resemble a Helmholtz resonator [42]. Transmission loss—that is, a sharp decrease in sound pressure down the air column—was considered indicative of a Helmholtz resonance and was calculated as the average pressure (Pa) below the apertures (distal) relative to that of the proximal input above (figure 7*d*).

### 2.5. Data analysis

Thickness results are reported as mean ± s.d. Normality was assessed using the Shapiro–Wilk test on residuals. Homogeneity of variances was tested using Levene’s test. Data were assumed to be normally distributed and with homogeneity of variances, therefore a one-way analysis of variance (ANOVA) with Tukey’s HSD *post-hoc* test was performed. Significance was defined as *p* < 0.05. Statistical analysis was performed in R (v. 3.6.1). Vibrometry results are reported as mean ± s.e.m. and only data points above 85% coherence were kept. FRFs were smoothed by a moving average filter of five frequency bins.

## 3. Results

### 3.1. Evidence of DivM functionality according to µ-CT 3D analysis

Thickness colour maps highlighting <10 µm cuticle in red indicated the membrane between the branches—the DivM—to be (i) within the same thickness range as the DM-PTB and PTM, and (ii) in a coupling arrangement with these membranes (figure 2*d,g* [Fig F2] [Fig F2] [Fig F2] and electronic supplementary material, animation S1). The DivM was also conspicuous under light microscopy (figure 2*e,f*). Average thickness values from the subsequent 3D analysis of five segmentations (figure 1*b,c*) were: PTM, 6.8 ± 1.2 µm; DM-PTB, 5.2 ± 2.1 µm; DivM, 6.2 ± 1.2 µm; VW-ATB, 9.3 ± 1.6 µm; VW-PTB, 8.8 ± 2.5 µm (*n *= 12, mean ± s.d.). Statistical analysis showed the DivM to be significantly thinner than two of the five structures, the VW-ATB (***p<0.001) and the VW-PTB (**p<0.01) (figure 2*h*).

### 3.2. The PTM response according to LDV: evidence of DM-PTB tuning

#### 3.2.1. Two distinct peaks in the PTM vibrational response

##### 3.2.1.1. Acoustic condition 1

Recordings of PTM vibrations under 78 dB pure tones yielded two distinct mean-average (*n *= 10) velocity peaks, one at 5.8 kHz and the other at 13.8 kHz (see figure 3*a* [Fig F3] [Fig F3] [Fig F3] [Fig F3] [Fig F3]). Across individuals, the position of peak 1 ranged from 5.4 to 6.0 kHz while the position of peak 2 was more variable with a range of 12.2–16.0 kHz.

##### 3.2.1.2. Acoustic condition 2

Frequency sweeps revealed similar results: a clear mean-average (*n *= 20) peak at 6.0 kHz and another at 14.3 kHz (see figure 3*c*).

Distinct peaks were also apparent from *T. commodus*, with the first at 6.4 kHz and the second at 12.2 kHz (mean, *n *= 6) (figure 3*e*).

### 3.2.2. Driving-force and natural resonance identified from PTM phase

PTM phase responses revealed that, at the 6 kHz vibrational peak (figure 3*a,c*), the tympanum was oscillating *in-phase* relative to the wavefront of the stimulus. This can be seen in figure 3*d* (mean, *n *= 20) as the real and imaginary plots (blue and orange, respectively) trending together at the 6 kHz position.

An in-phase response is characteristic of a resonant system which remains dominated by its mass momentum, necessarily below its natural resonance peak. This means that whatever was generating the PTM peak at 6 kHz was a *driving-force* external to the tympanum itself.

The position of this driving-force matches well with the PTM vibrations from both acoustic conditions 1 and 2, as highlighted by a yellow bar (see figure 3*a–d*). The width of the yellow highlight corresponds to the 5.9–6.2 kHz range across individuals (*n *= 20).

In contrast, at the second vibration peak near 14 kHz (figure 3*a,c*), the phase of the membrane sharply diverges. This is seen in figure 3*d* (mean, *n *= 20) as the blue and orange plots trending away from each other at the 14 kHz position. This *out-of-phase* response is characteristic of the *natural resonance* of a membrane.

The position of the PTM natural resonance is highlighted by a green bar. Its considerable width corresponds to the 11.2–19.3 kHz variability between individuals (*n *= 20). For example, the specimen of figure 3*b* exhibited a PTM natural resonance at 16.2 kHz.

From *T. commodus*, the phase responses (mean, *n *= 6) of the tympana likewise indicated that peak 1 (6.4 kHz) was a driving-force, while peak 2 (12.2 kHz) was the natural resonance of the tympanum (figure 3*e,f*).

The mathematical theory explaining the difference between a driving-force and the PTM natural resonance is provided in electronic supplementary material, appendix S4.

### 3.2.3. Effect of closing spiracles on PTM filtering

#### 3.2.3.1. Acoustic condition 3

Conceivably, the 6 kHz driving-force was driven by the signal via the auditory spiracles. Accordingly, we measured tympanal responses with and without the spiracles blocked (see figure 3*g*). The data, however, appear considerably noisy, at least from around 6 kHz upwards.

Yet, despite the noise, the corresponding phase data at least for one individual suggested a maintained 6 kHz driving-force after the spiracles had been blocked (see figure 3*h*, yellow highlight).

Overall, the data were not deemed suitably reliable and as such the effect of closing the spiracles was largely determined from the literature (see §4).

### 3.2.4. PTM modal response

The PTM oscillated with a fundamental (0,1) drum mode at all frequencies. This can be seen in figure 3*i–l* in the maximal velocities of each scan point at peaks 1 and 2 for both species (see also electronic supplementary material, animation S5). This means that only one of the two observed vibrational optima can have been the natural resonance of the membrane [35].

### 3.2.5. Candidate driver of the driving-force

Peak 1 at 6 kHz was from a driving-force and was therefore sourced from an external driver to the PTM. The obvious candidate driver was the DM-PTB membrane, which is known from the literature [18] and our µ-CT reconstructions (e.g. figure 2*g*) to be connected to the PTM at approximately 90° on its dorsal aspect.

### 3.3. Coupled resonators: demonstration of LDV results from FEA

The capacity for a resonator coupled to the tympanum to drive a driving-force peak on the PTM was demonstrated by numerical modelling (see figure 4). Only when the PTM was coupled did the PTM oscillate with a driving-force (figure 4*a*, yellow highlight).

### 3.4. A chain of coupled membranes: FEA

The DivM was shown from µ-CT to be a candidate coupled resonator (figure 2), as was the intermediate DM-PTB from LDV experiments (figure 3). How these membranes influence the complete system, from the PTM to the ATB supporting the sensilla, was numerically modelled (figure 5 [Fig F5] [Fig F5] [Fig F5] [Fig F5] [Fig F5]).

#### 3.4.1. Vibrational tuning of four membranes

The calculated vibrational profile of the PTM (figure 5*a*) showed robust agreement with experimental data (see figure 3*a,c*), in that the simulated tympanum responded with a dominant resonance at 10.7 kHz and a lower-frequency peak at 7.2 kHz.

In line with the DM-PTB functioning as the resonant driver of peak 1, the 7.2 kHz PTM peak exactly correlated with a principal sharp resonance of the DM-PTB (see figure 5*b*).

In agreement with the µ-CT investigation pointing to a DivM function, the simulated DivM vibrated with a dominant resonance at 7.0 kHz (figure 5*c*). Moreover, the DivM also exhibited a torque multiplication of the vibrational energy it received from the PTM: at the tympanum’s dominant frequency position, the DivM amplified the force 34-fold (see figure 5*e*). (For a theoretical explanation of strain energy, see electronic supplementary material, appendix S4.)

At 5.1 kHz, closer to the calling song CF, the dorsal membrane of the ATB supporting the sensilla vibrated with a distinct resonance (figure 5*d*), suggesting the possibility of *successive filtering*: 10.7 kHz PTM to 7.2 kHz DM-PTB to 5.1 kHz DM-ATB.

The 5.1 kHz DM-ATB resonance was correlated with an extremely faint but nonetheless evident disturbance in PTM vibrations (see figure 5*f*, left-hand star). A possible corresponding subpeak at 4.2 kHz may have been evident in the LDV experimental recordings (figure 5*g*, left-hand star; see also figure 3*a–f*).

#### 3.4.2. Membrane-mediated ATB volume change

The volume of the anterior branch across frequencies was calculated. The ATB exhibited volume fluctuations from 4.8 to 10.4 kHz (see figure 6*a* [Fig F6] [Fig F6] [Fig F6] [Fig F6] [Fig F6]). Intermediate peaks of volume increase corresponded to nulls (highlighted) at 5.1 and 7.1 kHz which matched the resonances of connected membranes: the DM-ATB, and the DivM and DM-PTB, respectively (figure 5*b–d*). The greatest increase in ATB volume occurred at 10.4 kHz, correlated with the tympanum’s dominant resonance (figure 5*a*).

If functioning as a resonant membrane, the DivM appears to be well-positioned at the interface of the ATB to possibly contribute to an ATB volume change (e.g. see figure 2*g*). As such, ATB volume was calculated *with* (teal) and *without* (black) the DivM in place (figure 6*a*). Without the DivM, there was a 24.5% loss in ATB volume at the maximal peak and all peaks shifted down by approximately 600 Hz.

Figure 6*b* shows the modelled ATB at different frequencies with boundaries colour-coded according to displacements as the ATB inflated and deflated. (Note that the rest of the model is not shown and that the full model was simulated intact, *with* the DivM in place.) The ATB was in a state of relative deflation at 2 kHz but increased in volume towards 4.7 kHz, with then a slight decrease, before expanding to maximal volume around 10 kHz, and then again deflating towards 18 kHz. The white arrow represents the approximate positions of the low- (L) to higher-frequency (H) tuned auditory neurons, as based on Nishino *et al*. [18].

### 3.5. Air column pressure and resonance according to FEA

Oscillating the PTM of the shell model (figure 7*a*) with 1 Pa pressure resulted in a less than 10% *pressure change* inside the ATB relative to that applied (see figure 7*b*), indicating PTM movement has a negligible influence on tracheal air pressure.

Using a segmentation of the air column (figure 7*c*) and the spiracular input as the sole pressure source, a *cavity resonance* (calculated as transmission loss) was not reached until 70.3 kHz (figure 7*d*), considerably higher than the spectral compositions of the calling, rivalry and courtship songs [6,7,11].

## 4. Discussion

### 4.1. The dividing membrane

Previous studies have proposed the existence of an additional frequency filter [14–19,33] and an unknown transmission pathway [13,16,18,19,22] behind the large tympanum of the field cricket ear. Most anatomical investigations of this region have been limited to 2D sectioned material [3,43,44], potentially restricting a comprehensive understanding of the functional anatomy. A significant advancement, however, was made in the 2019 confocal microscopy study by Nishino *et al*. [18], which included a pioneering 3D reconstruction of the ear. In our investigation, we employed µ-CT imaging to conduct 3D visualization and thickness analysis of the Gryllinae PTM and internal structures.

The 3D thickness analysis reveals a potential auditory function of the DivM structure separating the tracheal branches. This functionality is indicated by (i) its linkage to the DM-PTB and PTM membranes (figure 2*g*) and (ii) its significant thinness (figure 2*h*).

Despite its potential importance, some of the key anatomical studies on the field cricket ear do not mention the DivM [3,18,45]. In our volumetric visualizations (e.g. figure 2*a,b*), we too initially overlooked this structure. Its coupling and resonating potential were recognized only after applying thickness colour maps (figure 2*d,g*). Subsequent light microscopy (figure 2*e,f*), statistical analysis (figure 2*h*) and numerical modelling (figure 5*c,e*) further supported this observation.

Previous attention to this structure has nonetheless been applied. In detailing the field cricket tracheal branches, Friedman [46] referred to their separation by '*the thin cuticle wall*'. This thin wall was defined by Larsen *et al*. [16] as *'the partition*' and these authors further proposed an auditory function, suggesting the partition might act as a pressure difference receiver like the posterior tympanum. Although the authors acknowledge such a function is unlikely, their hypothesis shows that the DivM has been recognized as a potential resonator.

Furthermore, it has long been observed that the solid–solid connection of the PTM to adjacent tracheal branches serves as a potential transmission pathway to the sensors [16,18,21,22]. Larsen *et al*. [16] suggested that transmission from the PTM *'most likely includes the tracheal walls of the PTB and ATB*', while Schneider *et al*. [21] described the PTM-PTB-ATB arrangement as a *'coupled system*'.

Evidence of PTM–tracheal coupling supporting a transmission pathway is here presented. µ-CT thickness analysis identifies the dividing membrane as a possible resonator (figure 2). The DivM is further demonstrated from µ-CT-based modelling to amplify PTM transmission (figure 5*e*), contribute to increased ATB volume (figure 6*a*) and resonate at a distinct frequency (figure 5*c*). These findings introduce a specific pathway within tracheal coupling facilitating both transmission and filtering, from the PTM to the DivM via the DM-PTB.

### 4.2. Towards resolving the vibrational profile of the PTM

The posterior tympanum is the essential first link of the transmission path in the field cricket ear [10,19,20,22] and clearly functions as a frequency filter [14,15,17,19–21,30–32]. As such, any attempt to understand filtering and transmission in this system must include an understanding of the vibrational profile of this structure.

Yet the exact filtering characteristics of the PTM have remained uncertain. Previous studies have indicated a single low-frequency resonance close to the calling song CF [19,31], while others have shown two distinct peaks [15,17,20]. Tympanal tuning at the CF of the calling song has been presented as sharp [31,32] and as broad [19,30]. Most recently, a single broad resonance with an optimum above the calling song CF has been suggested [14,21].

#### 4.2.1. Experimental findings

The LDV results presented here of the PTM vibrational response show a clear distinction between a 6 kHz peak and a second near 14 kHz (figure 3*a–d*). The presence of a low-frequency peak between 4 and 7 kHz is consistent with most studies [15,17,19,20,30–32], and the position of a second peak near 14 kHz is also in broad agreement with previous recordings [15,17,20].

The more recent finding by Lankheet *et al*. [14], of a single broad 6–8 kHz resonance, was from normalized data. Conceivably therefore, differentiation of distinct peaks may have been lost in the averaging. As for the 11–17 kHz optimum subsequently reported by Schneider *et al*. [21], this result used only one measurement below 7 kHz and therefore a 6 kHz first peak could not possibly have been recognized.

We found the tympanum oscillated at both 6 and 14 kHz with the same up-down drum mode (figure 3*i–l* and electronic supplementary material, animation S5). This result agrees with earlier observations [17,19] and means that only one of the two vibrational optima can be the natural resonance of the tympanum [35].

The natural resonance of the PTM was identifiable in the phase responses and was consistently positioned above 11 kHz (figure 3*a–d*, green highlight). In contrast, the 6 kHz peak was revealed to be the product of a driving-force, driven from an external source to the tympanum (figure 3*a–d*, yellow highlight). What then is the driver that is vibrating the tympanum at 6 kHz?

Conceivably, the 6 kHz optimum is driven by a phase-shifted signal from the auditory spiracles acting on the tympanum’s internal surface. However, in figure 3*h* the 6 kHz driving-force appears maintained in an individual with *blocked* spiracles. More compelling is the study by Larsen [15] in which both spiracles of *G. bimaculatus* were blocked and yet a sharp velocity peak near 6 kHz was observed, alongside a distinct higher-frequency optimum. Together, this evidence strongly indicates that the 6 kHz peak is not driven by the spiracular inputs.

Alternatively, the driver is a mechanical resonator vibrating at its own natural resonant frequency of 6 kHz that is somehow affecting the PTM. Strikingly, Larsen [15] hypothesized such a resonator in 1981: *'… the mechanical system of the ear responds almost as if it consisted of two coupled, simple oscillators, one with a high-frequency vibration, and another with a low-frequency vibration. The identity of the two hypothetical oscillators is still obscure …*'.

Based on the 2019 anatomical study by Nishino *et al*. [18] as well as our own µ-CT results (figure 2), the obvious candidate resonator is the dorsal membrane of the posterior branch. This membrane is known to be connected to the inside surface of the tympanum (figure 2*g*).

There are at least two reasons why the properties of the DM-PTB and not the PTM may be facilitating a lower-frequency 6 kHz resonance: (i) the DM-PTB is thinner, even if not significantly so (see figure 2*h*), and a membrane even slightly thinner will resonate at a considerably lower frequency due to its flexural rigidity being proportional to the *cubed* of its thickness (see electronic supplementary material, appendix S4). (ii) The cuticle of the field cricket leg trachea is known to contain more resilin than the PTM [41], and this would be expected to lower the Young’s modulus of the DM-PTB and therefore its resonant frequency.

#### 4.2.2. Numerical modelling

The conclusion from experimental results, that the DM-PTB is driving the 6 kHz peak of the PTM, is strongly supported by the numerical modelling:

The first FEA model demonstrated the action of a coupled resonator on the PTM phase response and showed that the driving-force was only evident on the PTM when it was mechanically coupled (figure 4*a*, yellow highlight). When uncoupled, the PTM no longer vibrated with the lower-frequency peak (figure 4*b*).

The second model constructed from µ-CT data (figure 5) simulated the vibrational velocities of both the PTM (figure 5*a*) and the DM-PTB (figure 5*b*). The DM-PTB vibrated with a sharp resonance that exactly coincided with a lower-intensity peak in the PTM response.

#### 4.2.3. The vibrational profile

Taken together, we can construct the probable profile of PTM vibrations in the field cricket: the posterior tympanum vibrates with two frequency optima, one its natural resonance of around 14 kHz, the other a 6 kHz driving-force from its coupling to the DM-PTB resonator.

### 4.3. Conclusions

#### 4.3.1. Summary of key findings

Here, a coupled resonance potential of the field cricket dividing membrane is revealed by 3D µ-CT thickness analysis and corroborated by numerical modelling. LDV recordings of the PTM confirm two vibrational optima with a natural resonance around 14 kHz and a driving-force at 6 kHz probably driven by the tuning of the internally coupled DM-PTB, a conclusion supported by FEA calculations. FEA further suggests a train of successive filtering that also includes the dorsal membrane of the ATB beneath the sensilla, with the ATB subject to a possible membrane-mediated volume change. Together, these findings indicate that in the field cricket ear both frequency filtering and transmission are performed at least in part by independently tuned mechanically coupled membranes.

#### 4.3.2. Coupled membranes and other ensiferans

The tracheal branches of the ensiferan ear have previously been thought to function mainly to channel sound in the air column to the tympana [24]. However, in 2021, the bushcricket (Tettigoniidae) ‘tracheal septum’, a structure that is analogous to the field cricket dividing membrane, was shown by optical coherence tomography (OCT) to move in-phase with the PTM, thus indicating its role in mechanically coupling transmission from the PTM to the sensory neurons [47]. In the same year, an OCT study on the tree cricket (Oecanthinae) acoustic trachea revealed similar results [24]. While the morphology of the field cricket tracheal branches differs from that of these ensiferans, our findings align with these recent studies and reinforce the importance of continuing to investigate how tracheal structures mechanically transmit and filter signals from the tympana to the auditory neurons.

#### 4.3.3. Future work

Unlike the bushcricket ear [48], the means of tonotopy in the field cricket remains undefined, although it has been suggested to involve a ‘travelling wave’ [33]. Applying OCT to the field cricket ear would provide direct measurements of its internal membranes. As would LDV recordings if the membranes could be exposed without compromising the system. Such measurements, especially of the DM-ATB, may elucidate a travelling wave if present.

An integral part of the field cricket’s importance as a neuroethology model [9] is its frequency tuning at each stage of the auditory pathway [33]. Continuing to clarify the biophysical source of this pathway may help to contextualize our understanding of frequency filtering at the neural and behavioural levels.

Lastly, a chain of microscale coupled membranes facilitating both filtering and transmission would be noteworthy. The field cricket ear is unusually small [18] and sharply tuned to low frequencies [1], while also capable of spectral decomposition [2]. The relevance of the membranes here described to micro-electromechanical system ‘MEMS’ diaphragm microphones—used in today’s smartphones and hearing aids [49]—is therefore apparent. Especially considering the ear of the parasitoid fly *Ormia ochracea* is also tuned to the field cricket’s calling song CF [50] and has already inspired microphone patents numbering today in double figures [51].

## Data Availability

Data supporting this article are deposited in the University of Strathclyde data repository and openly available at [52]. Electronic supplementary material is available online at [53].
